# The high expression of NUDT5 indicates poor prognosis of breast cancer by modulating AKT / Cyclin D signaling

**DOI:** 10.1371/journal.pone.0245876

**Published:** 2021-02-11

**Authors:** He Zhang, Li-Qun Zhang, Cheng-Cheng Yang, Jin Li, Xin-Yuan Tian, Dan-Ni Li, Ju Cui, Jian-Ping Cai

**Affiliations:** 1 Graduate School of Peking Union Medical College and Chinese Academy of Medical Sciences, Dongdan, Beijing, P.R China; 2 The Key Laboratory of Geriatrics, Beijing Institute of Geriatrics, Beijing Hospital, National Center of Gerontology, National Health Commission, Dong Dan, Beijing, P.R. China; 3 Institute of Geriatric Medicine, Chinese Academy of Medical Sciences, Dong Dan, Beijing, P.R. China; 4 Department of Pharmacy, Wenzhou Medical College, Wenzhou, Zhejiang, P.R China; 5 Peking University Fifth School of Clinical Medicine, Beijing Hospital, Beijing, P.R. China; Duke University School of Medicine, UNITED STATES

## Abstract

NUDIX hydrolase type 5 (NUDT5) is a kind of ADP-ribose pyrophosphatase and nucleotide metabolizing enzyme in cell metabolism. Previous studies have shown NUDT5 expression affected chromosome remodeling, involved in cell adhesion, cancer stem cell maintenance and epithelial to mesenchyme transition in breast cancer cells. Nevertheless, the role of NUDT5 in breast cancer progression and prognosis has not yet been systematically studied. This study explored the association of NUDT5 with the tumor development and poor prognosis in patients with breast cancer. Our results show that the levels of NUDT5 were upregulated in breast cancer cell lines and breast tumor tissues, and the expression of NUDT5 in breast tumor tissues increased significantly when compared with adjacent non-tumorous tissues by immunohistochemical staining of tissue microarrays. Breast cancer patients with high NUDT5 expression had a worse prognosis than those with low expression of NUDT5. In addition, the knockdown of NUDT5 suppressed breast cancer cell lines proliferation, migration and invasion, and dramatically inhibited the AKT phosphorylation at Thr308 and expression of Cyclin D1. The opposite effects were observed in vitro following NUDT5 rescue. Our findings indicated that the high expression of NUDT5 is probably involved in the poor prognosis of breast cancer via the activation of the AKT / Cyclin D pathways, which could be a prognostic factor and potential target in the diagnosis and treatment of breast cancer.

## Introduction

According to the World Health Organization (WHO), approximately one eighth of women have a lifetime risk of breast cancer [1, 2]. In United States, breast cancer alone accounts for 30% of all new cancer diagnoses in women in Cancer Statistics 2019 [3]. In Asia, South America, and Africa, the incidence of breast cancer is also increasing [4–6]. Breast cancer is still the most common cause of death in developing countries and its mortality rate is second to lung cancer in developed countries [7]. However, the pathogenesis of breast cancer is a complex process and remains unclear [8].

Poly adenosine diphosphate (ADP)-ribose polymerase inhibitors (PARPi) can effectively inhibit the growth of BRCA1 and BRCA2 mutant breast cancer [9–15]. PARPi elevate ADP-ribose (ADPR) in cells by inhibiting Poly adenosine diphosphate (ADP)-ribose polymerase (PARP) activity. ADPR is the metabolite of poly ADPR, cycle ADPR and NAD+. High level of ADPR can cause protein ADP-ribosylation and ADPR-dependent cell dysfunctions thus inhibit tumor cell growth [16–19]. Although the currently commonly used PARPi drugs have effects on BRCA-mutated breast cancer patients, traditional therapies still play a major role in the treatment of breast cancer [15].

Previous researches showed that NUDT5 was significantly correlated with nucleotide metabolism and cancer. NUDT5 is an ADP ribose pyrophosphatase, which can rapidly hydrolyze ADPR, regulate intracellular ADPR concentration, and maintain normal physiological activities of cells [20]. Recently, a research indicated that PARP and NUDT5 were both involved in a mechanism for energy generation in the nuclei. They exhibited that high NUDT5 expression increased adenosine triphosphate (ATP) synthesis in the nucleus and affected chromosome remodeling [20]. NUDT5 also involved in cell adhesion, cancer stem cell maintenance and epithelial to mesenchyme transition in breast cancer cells [21, 22]. NUDT5 also significantly correlate with nucleotide metabolism and colorectal tumor. Zhang et al. reported that low NUDT5 expression resulted in G1 phase cell cycle arrested in HeLa cells [23] and led to apoptosis and RNA oxidation in IMR-90 fibroblasts [24]. Li et al. demonstrated that NUDT5 was a well predictor of progression and a prognostic marker of colorectal tumor [25]. Therefore, NUDT5 may be not only an ADP ribose pyrophosphatase associated with PARP function but also a multi-functional hydrolase that contributes to cell growth. However, the relationship between NUDT5 and breast cancer have not yet been thoroughly studied.

In the present study, we investigate the expression of NUDT5 in breast cancer cell lines and specimens. We evaluated the association between the expression of NUDT5 and the clinicopathological features and survival of breast cancer patients. We explored the molecular mechanisms by which NUDT5 influence breast cancer. We also observed that knockdown NUDT5 proteins inhibited the growth and metastasis ability of cell. Finally, we explored the signaling pathways underlying breast cancer alteration.

## Materials and methods

### Patients and specimens

Tumorous and adjacent non-tumorous breast tissues were obtained from tissue microarrays (TMAs) containing 30 paired breast cancer specimens (median age, 54.5 years; 100% female) and 140 breast cancer specimens (median age, 55.9 years; 100% female) purchased from the Xin Chao Company (Shanghai Outdo Biotech Co., Ltd., Shanghai, China). The patients underwent surgery between January 2005 and September 2012 and were followed up until August 2016. The diagnosis of breast cancer was confirmed by histologic examinations. The tumor-node-metastasis (TNM) classification of our study was made according to the sixth edition of the American Joint Committee on Cancer (AJCC)/Union for International Cancer Control; 20 patients were graded as stage I–II and 10 patients as stage III–IV among the 30 paired breast cancer patients, and 94 patients were graded as stage I–II and 46 as stage III–IV among the 140 breast cancer patients.

### Cell lines and cell culture

The human breast cancer cell lines MCF-7, MDA-MB-231 and T47D were obtained from the Type Culture Collection of the Chinese Academy of Medical Sciences (Beijing, China). MCF-7 cells were grown in Dulbecco’s modified Eagle’s medium, MDA-MB-231 cells were grown in L15 medium, and T47D cells were grown in RPMI 1640 medium (containing 0.2 U/mL bovine insulin) supplemented with 10% fetal bovine serum (FBS; Gibco, USA). The cell lines were maintained at 37°C in a 5% humidified CO_2_ atmosphere.

### Quantification of NUDT5 expression by immunohistochemistry

NUDT5 expression in primary tumors and adjacent noncancerous breast tissue was examined using immunohistochemistry (IHC). TMAs (HBre-Duc060CS-03 and HBreD140Su03) were de-waxed using xylene, dehydrated with 100%, 90%, 80% and 70% graded ethanol washes, washed with phosphate-buffered saline, and microwaved in citric acid buffer (pH 6.0) at 95°C for 45 min. Endogenous peroxidase was consumed by 3% H_2_O_2_ for 15 min and blocked with 10% goat serum (ZSGB-BIO, Beijing, China), the TMAs were incubated at 4°C overnight in a dark box with the western blotting anti-NUDT5 antibody (1:1000 dilution). Other slides were incubated with goat serum instead of the primary antibody as a negative control. A secondary antibody was applied using the Polink-1 HRP 3,3′-diaminobenzidine (DAB) Detection System (ZSGB-BIO) for 20 min. the IHC visualized with a DAB kit (ZSGB-BIO), stained for 90 s with DAB and then counterstained with hematoxylin (ZSGB-BIO). A Nikon Eclipse 80i microscope (Nikon, Tokyo, Japan) was used to observe and photograph the images. All controls yielded satisfactory results.

Low and high expression of NUDT5 immunoreactivity was accorded to the intensity and proportion of immunostaining of the tissues. No staining intensity was scored as 0, weak staining intensity was scored as 1, moderate staining intensity was scored as 2 and strong staining intensity was scored as 3. The staining proportion of the breast tissue cells was scored as 0 (0%), 1 (1–25%), 2 (26–50%), 3 (51–75%), and 4 (76–100%). The final IHC score was calculated by multiplying staining intensity by staining proportion, with 0–6 considered as low expression and 7–12 as high expression [14].

### RNA interference and transfection

pSIREN-RetroQ-shNUDT5 and pSIREN-RetroQ-shScramble plasmids were constructed in a previous study [12]. Small interfering RNA targeting NUDT5 (5′-CATGGATCCTACTGGTAAA-3′) and the negative control were synthesized by RiboBio Co., Ltd. (Guangzhou, China).

For transfections, MCF-7 and T47D cells were seeded into 24-well plates at 60–70% confluence. After 24 h, the cells were transfected with pSIREN-RetroQ-shNUDT5 and pSIREN-RetroQ-shScramble plasmids using Lipofectamine 3000 (Invitrogen, Carlsbad, CA) according to the manufacturer’s instructions. After 8 h of incubation at 37°C, the transfection medium was replaced with 500 μL complete medium containing 10% FBS and 1.2 μg/mL puromycin. The MDA-MB-231 cells were unable to survive transfection using this approach, so for transient transfections, MDA-MB-231 cells were seeded into 24-well plates at 60–70% confluence. After 24 h, the cells were transfected with RNAiMAX (Life Technologies, USA) according to the manufacturer’s instructions. These cells were collected for quantitative real-time polymerase chain reaction (qRT-PCR), western blotting, and cell viability, migration, and invasion assays at the indicated times.

### Rescue assay

NUDT5 and control sequences were amplified from human gDNA with site directed mutagenesis and then cloned into the retroviral vector pLVX-CMV. Packaging was performed in 293T cells cultured in DMEM with 10% FBS in a 37°C incubator with 5% CO_2_. 48 hours after transfection, the supernatant was harvested and cleared by centrifugation at 1,000 g for 10 minutes. MCF-7-shNUDT5 and MCF-7-shScramble cells were then transduced with the retrovirus containing NUDT5 or control plasmids. 72 hours after infection, 1000 mg/mL G418 was added to the medium for 2 weeks to select retrovirus-infected cells. Western blotting was performed to evaluate NUDT5 expression in a stable cell line.

### qRT-PCR

For quantitative reverse transcription-PCR (qRT-PCR), TRIzol reagent (Thermo Fisher Scientific, Boston, USA) was used to isolate the total RNA from cell lines, and 100ng RNA in total volume of 25μl mix in One Step SYBR^®^ PrimeScript^™^ RT-PCR Kit (TAKARA, Tokyo, Japan). Each reaction was performed in triplicate. The relative mRNA expression of NUDT5 genes was normalized to that of GAPDH. The primer sequences used for qPCR were as follows: NUDT5, forward 5’-GTTCTCCAGCGGTCTGTATG-3’ and reverse 5’-CTTCGGCCTTGCGTTTTCG-3’; GAPDH, forward 5’-CCTCTCCAGAACATCATCC-3’ and reverse 5’-GTGTCGCTGTTGAAGTCAG-3’. The experiment was repeated three times.

### Western blotting

Total protein was extracted by RIPA lysis buffer (Solarbio, Beijing, China) which containing 1× PMSF (Solarbio) and 1× Protease/Phosphatase Inhibitor Cocktail (Cell Signaling Technology, Beverly, MA), from the cell lines. BCA Protein Assay Kit (Thermo Fisher Scientific, Waltham, MA) was used to measure protein concentrations then diluted them to 2 μg/μl by the 6× loading buffer and RIPA lysis buffer and denatured at 100°C for 7 min. Protein lysates (20 μg) were resolved by 12% sodium dodecyl sulfate-polyacrylamide gel electrophoresis and transferred electrophoretically onto a polyvinylidene fluoride membrane. After blocking for nonspecific binding, the blots were incubated with specific antibodies against NUDT5 (ab129172; Abcam, Cambridge, UK; 1:1000 dilution), extracellular signal-regulated kinase (ERK)1/2 (137F5; Cell Signaling Technology; 1:1000 dilution), phosphorylated (p)-ERK1/2 (T202, Y204, D13.14.4E; Cell Signaling Technology; 1:1000 dilution), protein kinase B (AKT) (pan, C67E7; Cell Signaling Technology; 1:1000 dilution), p-AKT (T308, D25E6; Cell Signaling Technology; 1:1000 dilution), Cyclin D1 (A11022; ABclonal, Wuhan, China; 1:1000 dilution), and β-actin (AC026; ABclonal; 1:10000 dilution). After incubation with a horseradish peroxidase (HRP)-conjugated secondary antibody (Beijing Biofriend Biotechnology Co., Ltd., Beijing, China), Protein bands were detected using Tanon V8 (Tanon, Shanghai, China) and imaging data were quantified using ImageJ software (National Institutes of Health, Bethesda, MD). The experiment was repeated three times.

### Cell viability assay by cell counting Kit-8

Transfected MCF-7 and T47D cells were seeded into 96-well plates at 1.0 × 104 cells/well, and MDA-MB-231 cells were seeded into 96-well plates at 5.0 × 103 cells/well. After 24, 48, and 72 h of incubation, 10μl Cell Counting Kit-8 (CCK-8) reagent (Dojindo Molecular Technologies, Tokyo, Japan) and 90μl complete medium mixture was added to each well, and the plates were incubated at 37°C for 1h. Cell viability is detected absorbance of samples at 450 nm using a Tecan Genios microplate reader (TECAN, Grodig, Austria). The results were represented mean ± standard deviation (SD) from detection of six wells for each group at indicated time points in MCF-7, T47D and MDA-MB-231 cells.

### Migration and invasion assay

Transfected MCF-7 (1.0 × 10^5^ cells), T47D (1.0 × 10^5^ cells), and MDA-MB-231 (5.0 × 10^4^ cells) cells were serum-starved in FBS free mediums overnight and then seeded into 24-well plates with or without 1.2 mg/mL Matrigel (354234; Corning USA) coated inserts (8-mm pore; BD Falcon, San Jose, CA). Mediums in the upper inserts with 5% FBS and 20% FBS mediums were added into 24-well plates. After 48 h, the cells attached to the lower surface of the insert filter were counted following crystal violet staining. The experiment was repeated three times.

### Statistical analysis

Statistical analyses were performed using SPSS version 20.0 software (SPSS, Inc., Chicago, IL). Significant associations between NUDT5 expression and clinicopathologic parameters were assessed using the χ2 test. Kaplan-Meier and log-rank tests were used to compare patient survival and to create survival curves based on high and low NUDT5 scores. Multivariate survival analysis was performed for all parameters that were significant in univariate analyses using the Cox regression model. Student’s t-test was used to compare the means from two groups. In this study, a P-value < 0.05 was considered to indicate statistical significance.

## Results

### High expression of NUDT5 detected in human breast cancer specimens

**Fig 1:**
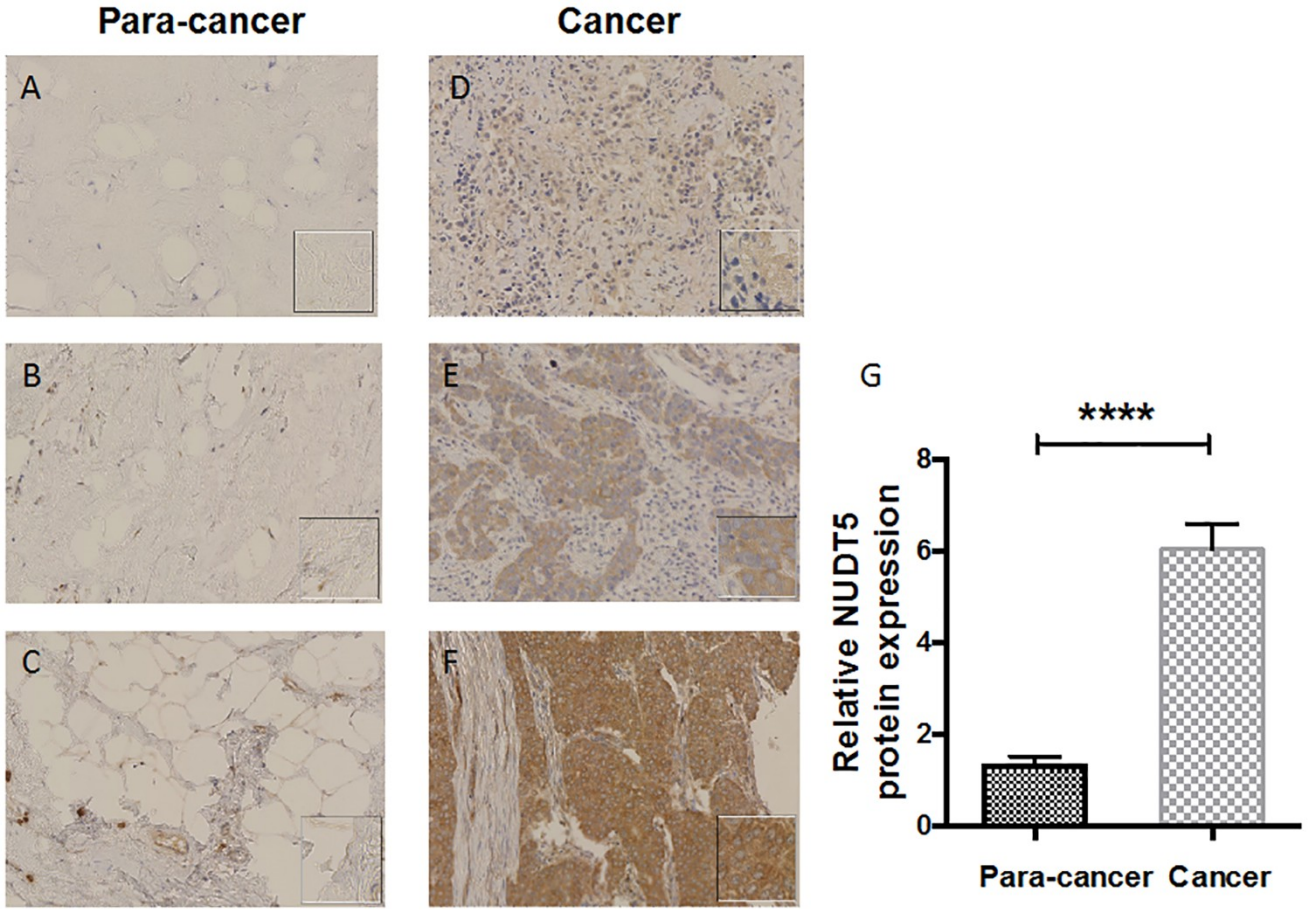
Representative IHC staining (200×) of NUDT5 expression in 30 breast cancer specimens. A–C, Very low NUDT5 expression in para-cancer tissues. D, Low NUDT5 expression in tumor tissues. E, Medium NUDT5 expression in tumor tissues. F, High NUDT5 expression in tumor tissues. The tissues of A and D, B and E, and C and F were obtained from each of 3 patients. G, NUDT5 protein expression was examined by IHC in 30 paired breast cancer and para-cancer tissues (P < 0.0001, Student’s t-test).

IHC analysis of TMAs showed that the protein of nudt5 was highly expressed in tumor tissue. The clinical details of the specimens were listed in Table 1. There are different degrees of positive staining in tumor tissue, that is, weak, medium and strong (Fig 1). The immunostaining of para-cancer tissues is weak. NUDT5 was found to be upregulated in tumors compared with paired adjacent non-tumor tissues. High NUDT5 expression was observed in 22 of 30 breast cancer specimens compared to all the para-cancer tissues specimens (Table 2).

**Table 1:** Patient and tumor characteristics.

Characteristics		Number of patients (N = 30)	%
**Average age (years)**	54.48		
**Grade**			
	I	0	0.00
	II	20	66.67
	III	10	33.33
**T**			
	1	8	26.67
	2	14	46.67
	3 + 4	8	26.67
**N**			
	0	13	43.33
	1	17	56.67
**M**			
	0	0	0.00
	1	0	0.00
**Lymphatic vessel invasion**			
	YES	10	33.33
	NO	20	66.67

**Table 2:** NUDT5 expression in breast cancer and para-cancer tissues.

	Breast Cancer		Expression ratio %	Para-Cancer Tissues		Expression ratio %	*P* value
	Positive	Negative		Positive	Negative		
**NUDT5**	22	8	73.3333	0	30	0	<0.0001*

*: Statistically significant.

### NUDT5 high-expression associated with decreased survival in human breast cancer

**Fig 2:**
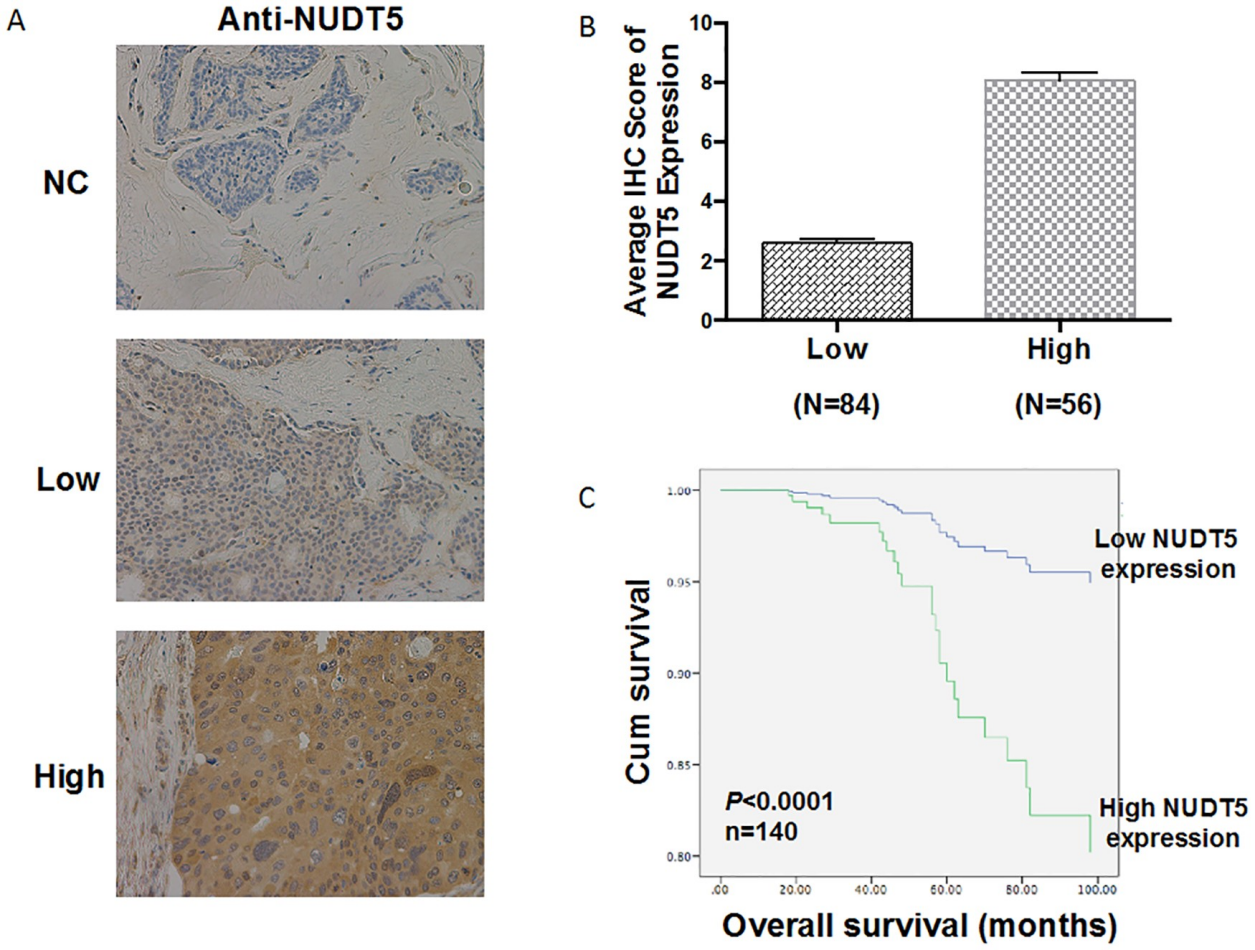
NUDT5 expression in 140 breast cancer tissues and its prognostic value. A, Representative IHC images showing the negative, low, and high expression of NUDT5 in breast cancer tissues (200×). B, Average IHC score of NUDT5 expression was examined in 84 breast cancer tissues (low expression) and 56 breast cancer tissues (high expression). C, Kaplan-Meier survival analysis according to IHC score of NUDT5 expression in patients with breast cancer (log-rank test).

We investigated the association between NUDT5 expression and the pathologic characteristics of 140 breast cancer samples (Table 3) by performing IHC. The survival time of the patients ranged from 39.6 to 132 months. Representative IHC staining images with NUDT5 were shown in Fig 2A. High NUDT5 expression was observed in 56 of 140 breast cancer specimens (Fig 2B). Pearson’s analysis showed that high NUDT5 expression was associated with age (P = 0.021), N stage (P = 0.013), recrudescence (P = 0.002), human epidermal growth factor receptor 2 (Her-2; P = 0.036) and estrogen receptor (ER; P = 0.016) (Table 3).

**Table 3:** Clinicopathologic findings and correlation with the NUDT5 expression (n = 140).

	N (%)	NUDT5-Low (%)	NUDT5-High (%)	*P*-value
**Total cases**				
**Age, years**				0.021*
≤55	78	54	24	
>55	62	31	31	
**Sex**				
Male	0	0	0	
Female	140	85	55	
**Tumor location**				0.138
Left	72	48	24	
Right	68	37	31	
**AJCC stage**				0.040*
I–II	94	62	32	
III–IV	46	23	23	
**TNM stage**				
**T stage**				0.253
T1–T2	138	83	55	
T3–T4	2	2	0	
**N stage**				0.013*
N0	75	52	23	
N1	19	10	9	
N2	38	22	16	
N3	8	1	7	
**Lymphatic vessel invasion**				0.351
No	124	77	47	
Yes	16	8	8	
**Recrudescence**				0.002*
No	102	70	32	
Yes	38	15	23	
**Preoperative Her-2**^#^				0.036*
(-)	61	34	27	
(+)	58	43	15	
**Preoperative ER**^#^				0.016*
(-)	42	19	23	
(+)	54	23	31	
**PR**^#^				0.237
(-)	65	36	29	
(+)	72	47	25	
**E-cad**^#^				0.104
(-)	45	22	23	
(+)	50	23	27	
**HCK**^#^				0.764
(-)	49	29	20	
(+)	19	12	7	
**SMA**^#^				0.901
(-)	29	22	7	
(+)	24	11	13	
**S-100**^#^				0.656
(-)	15	10	5	
(+)	35	21	14	
**P53**^#^				0.437
(-)	66	38	28	
(+)	17	8	9	
**Bcl-2**^#^				0.632
(-)	26	14	12	
(+)	18	11	7	
**CK7**^#^				0.22
(-)	1	0	1	
(+)	41	25	16	

*: Statistically significant.

^#^: Several data points from the 140 cases were not collected.

Kaplan-Meier analysis with a log-rank test was performed to assess the relationship between NUDT5 expression and the overall survival (OS) of breast cancer patients. The group of NUDT5 high-expression had worse OS than those with low expression (P < 0.0001) (Fig 2C). As shown in Table 4, univariate analysis with a Cox proportional hazards model revealed that OS was associated with the following characteristics: AJCC stage (P = 0.0005), lymph node metastasis (P = 0.0043), recrudescence (P < 0.0001), ER expression (P = 0.0012), P53 expression (P = 0.0232) and NUDT5 expression (P < 0.0001). however, multivariate analysis indicated that high NUDT5 expression was an independent prognostic factor for the OS of patients with breast cancer (hazard ratio [HR] 0.114; 95% confidence interval [CI] 0.021–0.612; P = 0.0113).

**Table 4:** Univariate and multivariate analyses of the overall survival.

Factor	Examination object	Comparable object	Univariate analysis			Multivariate analysis		
			HR	95% CI	*P*-value	HR	95% CI	*P*-value
**Age, years**	>55	≤55	0.480	0.225–1.026	0.0583	0.671	0.182–2.469	0.5479
**Sex**	Female	Male						
**Tumor location**	RIGHT	LEFT	1.132	0.539–2.380	0.7431			
**AJCC stage**	III-IV	I-II	0.262	0.122–0.560	0.0005*	5.901	0.689–50.499	0.1051
**Tumor depth**	T3-T4	T1-T2	20.581	0.000–12417080.093	0.6561			
**Lymph node metastasis**	N (+)	N (−)	0.302	0.133–0.687	0.0043	0.123	0.013–1.193	0.0706
**Lymphatic vessel invasion**	YES	NO	0.528	0.201–1.389	0.1955			
**Recrudescence**	YES	NO	0.057	0.023–0.142	<0.0001*	0.100	0.019–0.536	0.0072*
**Preoperative Her-2**	(+)	(-)	2.388	0.990–5.759	0.0526	1.579	0.439–5.672	0.4840
**Preoperative ER**	(+)	(-)	3.450	1.631–7.296	0.0012*	4.533	1.155–17.782	0.0302*
**PR**	(+)	(-)	1.447	0.685–3.060	0.3330			
**E-cad**	(+)	(-)	0.967	0.453–2.064	0.9304			
**HCK**	(+)	(-)	0.861	0.233–3.182	0.8225			
**SMA**	(+)	(-)	0.375	0.081–1.737	0.2098			
**S-100**	(+)	(-)	1.206	0.243–5.975	0.8189			
**P53**	(+)	(-)	2.957	1.159–7.540	0.0232*	0.6340	0.202–1.992	0.4356
**Bcl-2**	(+)	(-)	0.650	0.163–2.602	0.5431			
**CK7**	(+)	(-)	0.330	0.042–2.557	0.2883			
**NUDT5**	High	Low	0.110	0.042–0.290	<0.0001*	0.1140	0.021–0.612	0.0113*

*Statistically significant.

### Inhibition of the growth, migration, and invasion in breast cancer cell lines by Knocking down of NUDT5 expression in vitro

**Fig 3:**
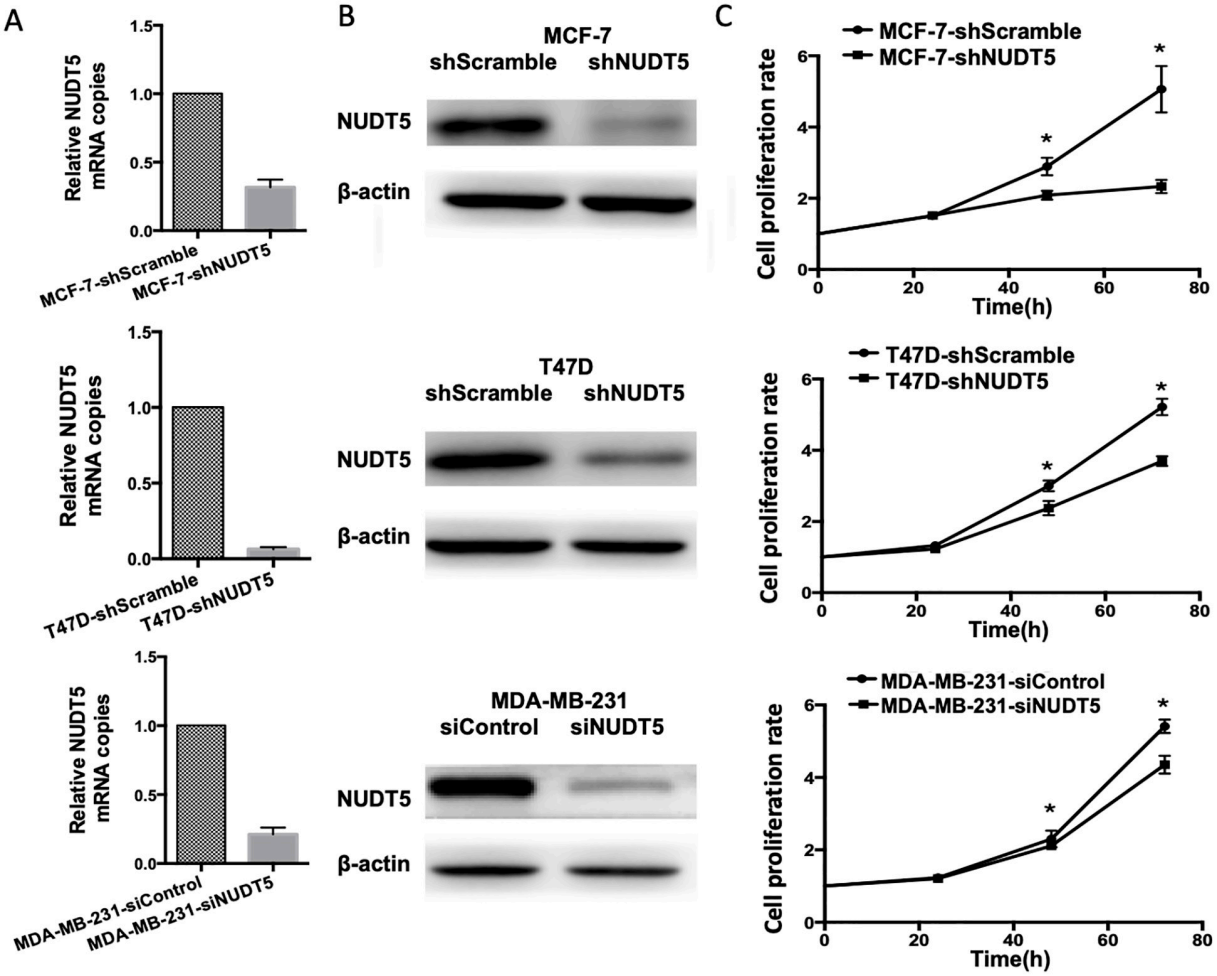
NUDT5 knockdown reduces the proliferation of MCF-7, T47D, and MDA-MB-231 cells. A, NUDT5 mRNA analysis with qRT-PCR. B, NUDT5 protein analysis with western blotting. C, Effects of NUDT5 knockdown on cell proliferation as detected by a CCK-8 assay. Student’s t-test, *P < 0.05.

Three typical cell lines were used to assess the role of NUDT5 in the proliferation, migration, and invasion of breast cancer cells. We knocked down NUDT5 in the MCF-7, MDA-MB-231, and T47D cell lines using short hairpin RNA targeting NUDT5 (shNUDT5) and evaluated knockdown efficiency by qRT-PCR and western blotting (Fig 3A and 3B). The impact of shNUDT5 on cell proliferation was evaluated with a CCK-8 assay. In MCF-7, MDA-MB-231 and T47D cell lines, nudt5 significantly inhibited cell proliferation at 72, 36 and 36 h, respectively (Student’s t-test, P < 0. 05, Fig 3C).

**Fig 4:**
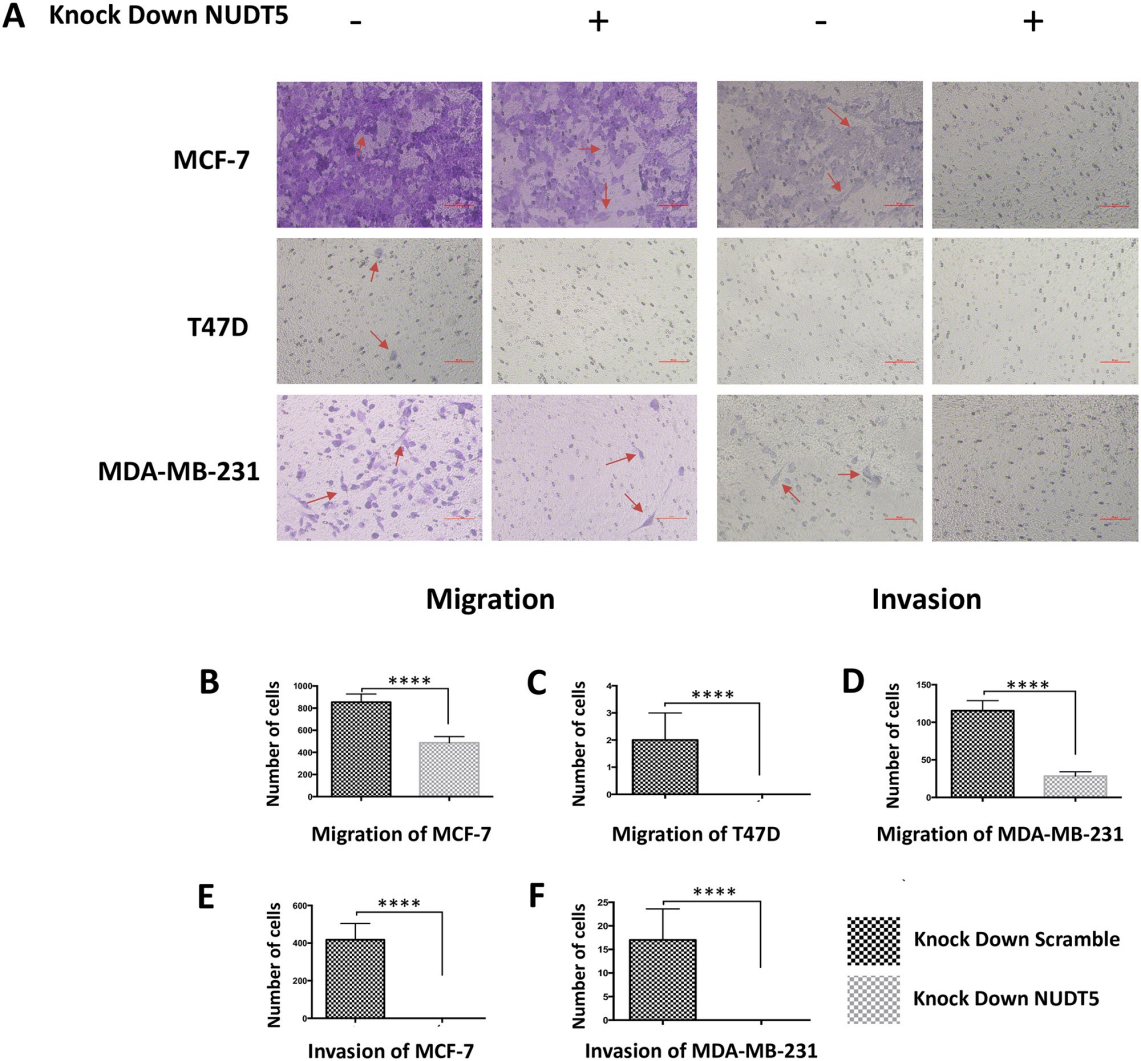
Low NUDT5 expression inhibits the migration and invasion of MCF-7, T47D, and MDA-MB-231 cells. The invasion of the MCF-7, MDA-MB-231, and T47D cell lines was reduced by NUDT5 knockdown. The migration of the MCF-7 and MDA-MB-231 cell lines was inhibited while migration was not detected in the T47D cell line.

The effects of shNUDT5 on cell invasion were determined using Transwell and Matrigel invasion assays. The migration of the MCF-7, MDA-MB-231, and T47D cell lines was reduced by NUDT5 knockdown (Student’s t-test, P < 0.001). The invasion of the MCF-7 and MDA-MB-231 cell lines was inhibited (Student’s t-test, P < 0.001) while invasion was not detected in the T47D cell line [26] (Fig 4).

### The inhibition of AKT phosphorylation and Cyclin D1 expression in breast cancer cells by Knocking down of NUDT5

**Fig 5:**
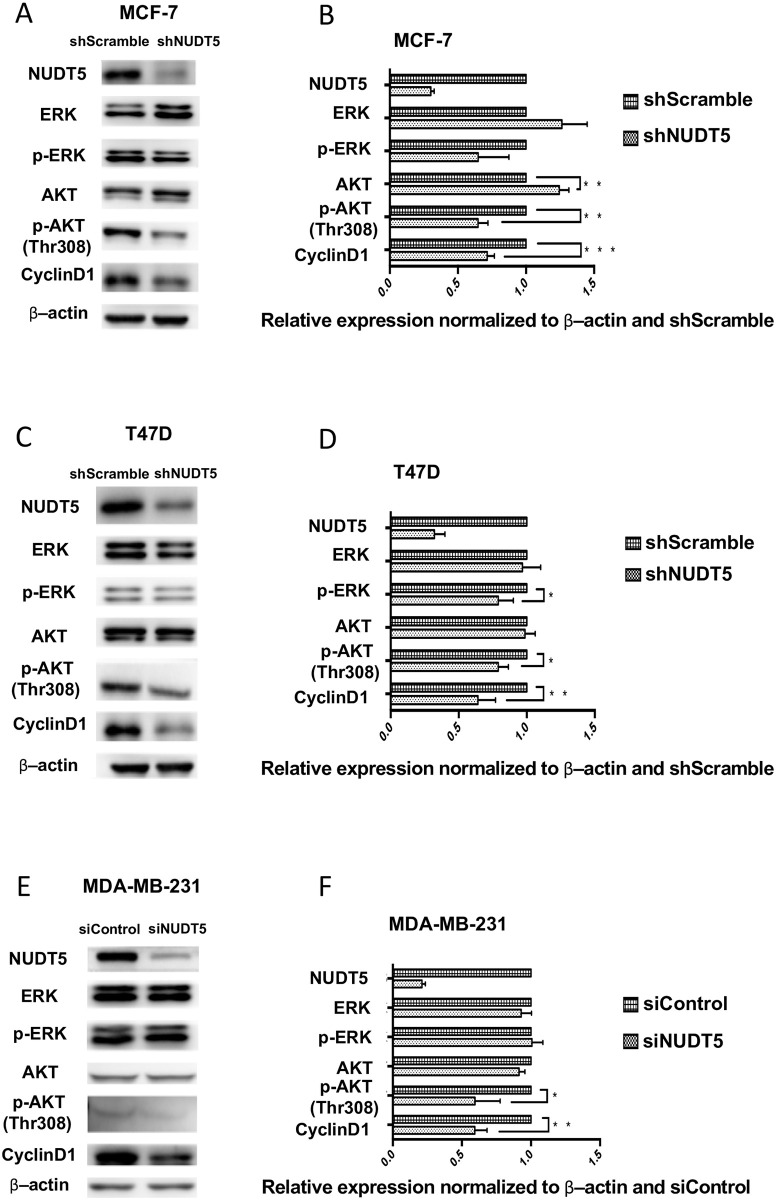
The inhibition of AKT phosphorylation and Cyclin D1 expression in breast cancer cells by Knocking down NUDT5. A, Representative images of MCF-7 and T47D cells with shNUDT5/empty vector (shScramble) and MDA-MB-231 cells with siNUDT5/siControl NUDT5. The expression of AKT, p-AKT, ERK, p-ERK, and cyclin D1 was analyzed by western blotting. B–D, Quantified expression of NUDT5, AKT, p-AKT, ERK, p-ERK, and cyclin D1 normalized to β-actin and scramble/negative control in MCF-7, T47D, and MDA-MB-231 cells. *P < 0.05, **P < 0.01, ***P < 0.001.

AKT and ERK activation was examined when NUDT5 expression was efficiently knocked down by shRNA in MCF-7, T47D and MDA-MB-231cell lines. Lower NUDT5 expression was related to the decreased levels of AKT, p-AKT(Thr308) and cyclin D1, while the levels of ERK and p-ERK had no significant changes in MCF-7 cell. For T47D cell line, shNUDT5 expression reduced p-AKT(Thr308), p-ERK and Cyclin D1 levels. In the MDA-MB-231 cell line, the levels of p-AKT(Thr308) and cyclin D1 were decreased by inhibiting the expression of NUDT5 (Fig 5). ERK and p-ERK levels were not significantly changed. Phosphorylation of AKT at Thr308 and the expression of Cyclin D1 levels were reduced by low NUDT5 expression in 3 breast cancer cell lines. While, AKT phosphorylation at Ser473 is inhibited in T47D cell line but not in MCF7 and MDA-MB-231 cell lines (S1 Fig) Essentially, decreased NUDT5 results in decreased p-AKT(Thr308) and cyclin D1.

### Rescue of NUDT5 expression reverse the inhibition of cell growth, migration, invasion ability and signaling pathway in MCF-7 cell

**Fig 6:**
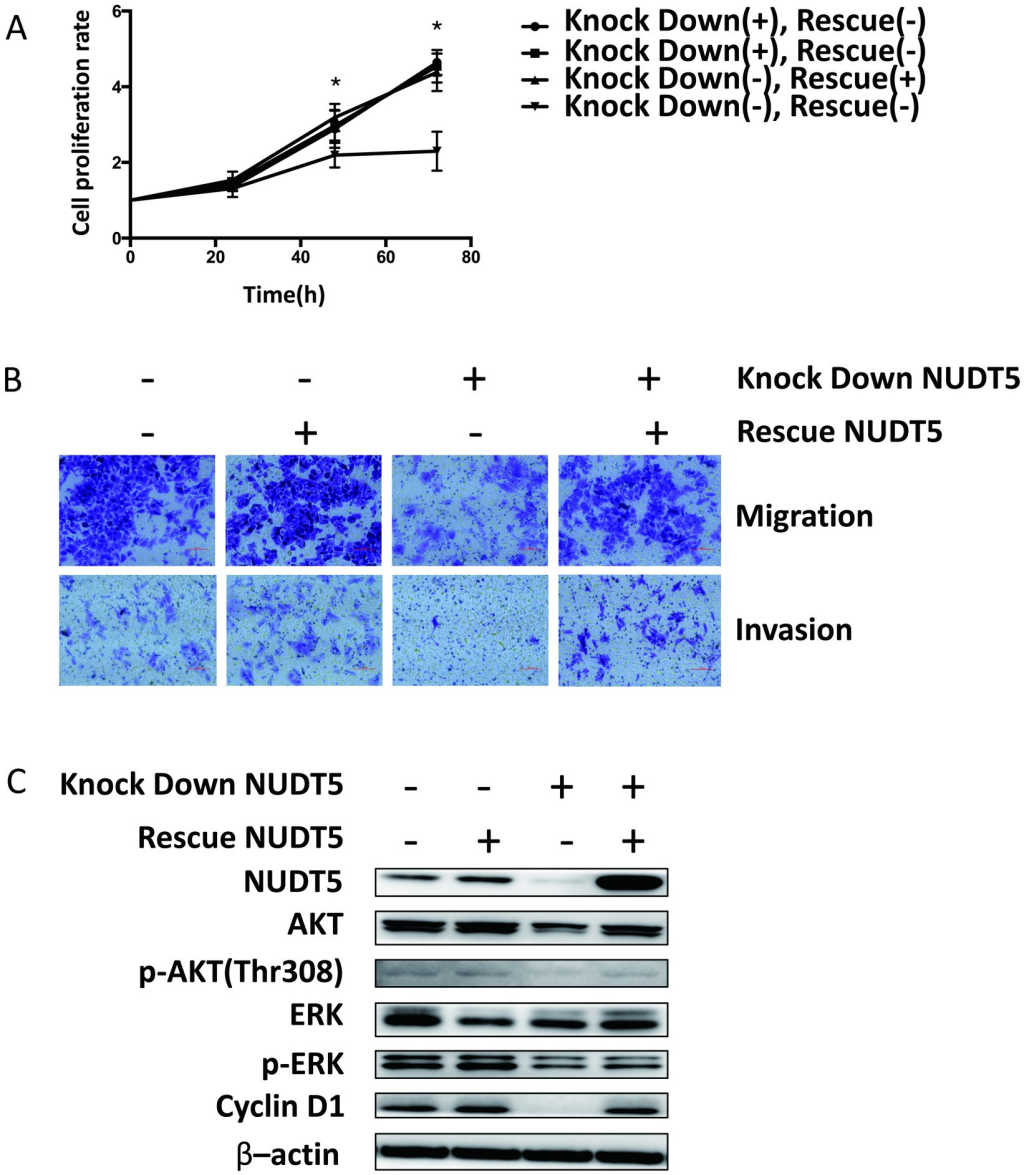
Rescue NUDT5 expression reverse cell growth, migration, invasion ability and signaling pathway in MCF-7 cell. Cell proliferation (A), invasion, migration (B) and activation of AKT and ERK (C) were all reinstated when re-expressing NUDT5 in NUDT5 knock down MCF-7 cell. Student’s t-test, *P < 0.05.

We evaluated Cell proliferation, invasion, migration and AKT and ERK activation by re-expressing NUDT5 in NUDT5 knock down MCF-7 cell. These cell functions were all reinstated (Fig 6).

## Discussion

Breast cancer has the highest morbidity in women in 140 countries, including both developed and developing countries (1). Previous studies have underscored the importance of early diagnosis and treatment in breast cancer (2). Due to the complexity of its pathogenesis, the mechanisms underlying the occurrence and development of breast cancer remain unclear.

As a member of the Nudix family with the specific Nudix box motif, its protein structure determines its ability to degrade ADP rapidly to adenosine monophosphate (AMP) for chromosome reconstruction (pH 7–9) in breast cancer cell lines [20, 27]. NUDT5 can also degrade 8-oxo-dGDP under alkaline conditions (pH 10) to 8-oxo-dGMP, which could not be converted to 8-oxo-dGTP, thereby preventing errors during DNA replication [28, 29]. A recent study identified TH5427 as a small-molecule inhibitor of NUDT5 [22]. We previously showed that NUDT5 knockdown leads to cell cycle arrest in HeLa cells and apoptosis in the IMR-90 cell line, and high NUDT5 expression is significantly correlated with the occurrence of colorectal cancer and its prognosis [23–25]. However, the role of NUDT5 in breast cancer remains unknown.

In our study, each cancer tissue and para-cancer tissue were matched individually. The results showed that NUDT5 was higher expressed in breast cancer tissues than in adjacent tissues. IHC of 30 paired breast cancer tissues at different pathological stages found that 100% of the para-cancer tissues (N = 30) had low NUDT5 expression, while 22 tumor tissues (73.33%) had high-expression of the NUDT5 (Student’s t-test; P < 0.001). Our results consistent with the previous study that found the expression of NUDT5 protein was lower in normal tissues than in tumor tissues, including breast cancer [30]. Furthermore, we found that patients with high NUDT5 expression had lower OS than those with low expression (P < 0.0001). In addition, univariate and multivariate analyses with a Cox proportional hazards model indicated that bad prognosis was associated with high NUDT5 expression (P < 0.0001 and P = 0.0113, respectively). These findings suggest that NUDT5 might be a potential prognostic factor in breast cancer.

Knocked down NUDT5 expression dramatically inhibited the proliferation, migration, and invasion of breast cancer cell lines in vitro. The AKT and ERK signaling pathways play important roles in cell growth, especially in tumor cells. The activation of AKT or ERK signaling enhances the biosynthesis ability of cells [31–35] and may lead to unlimited proliferation. Inhibition of ERK signaling benefits patients with abnormal activation of the RAS-RAF-MEK-ERK pathway (ERK signaling) [36]. The relationship between AKT and ovarian cancer [37], gastric cancer [38], pancreatic cancer [39], and hepatocellular carcinoma has been reported [40], and several relevant reports in the study of breast cancer have also been published [41]. In our studies, when the expression of NUDT5 was downregulated through RNA interference, the phosphorylation of ERK and AKT in MCF-7 and T47D cells and phosphorylation of AKT at Thr308 in MDA-MB-231 cells were reduced. These findings indicate that the decreased activation of ERK and AKT might be the result of the inhibition of NUDT5, which subsequently inhibited the proliferation, migration, and invasion of breast cancer cells. This assumption was further confirmed by rescuing NUDT5 expression in shNUDT5-MCF-7 cell.

It was noted that the level of Cyclin D1 was decreased significantly in the three cell lines when NUDT5 expression was inhibited. Cyclin D1 is a key protein for the promotion of the cell cycle from the G1 phase to the S phase. The inhibited expression of Cyclin D1 may lead to cell cycle arrest and tumor formation. Cyclin D1 overexpression is detected in a variety of tumors, especially in breast cancer [42]. Fu et al. [43] proposed that Cyclin D1 regulated the development and differentiation of mammary epithelial cells and Cyclin D1 overexpression leads to carcinogenesis in mammary epithelial cells. This view was proven in animal models [44]. Elsheikh et al. [45] showed in their clinical study that Cyclin D1 is highly expressed in the early stage of breast cancer and persists during the processes of invasion and metastasis, which are closely related to the expression of ER. Reis-Filho et al. [46] observed Cyclin D1 overexpression in 40–90% of invasive breast cancer samples by IHC and in situ hybridization. Cyclin D1 is an independent activator of ER and binds to its hormone-binding domain to activate ER; in this way, Cyclin D1 contributes to the development of ER-positive breast cancer [47].

In our study, we found the expression of Cyclin D1, cell proliferation, invasion and migration were decreased significantly when NUDT5 expression reduced. In our clinical data, NUDT5 expression was closely related to ER expression. The proportion of ER positivity was 30.65% in patients with low NUDT5 expression and 66.67% in those with high NUDT5 expression. On the basis of these findings, we speculated that Cyclin D1 expression was upregulated in NUDT5-highexpressing breast cancer tissues. The survival time of ER-positive patients with high NUDT5 expression might be significantly shorter than those with low NUDT5 expression. Cyclin D1 binds to the hormone-binding domain of ER, leading to the failure of ER-targeting drugs.

In breast cancer, NUDT5 overexpression not only increases ATP synthesis and reconstructs tumor cell chromosomes [20], it also activates the AKT pathways and increases the expression of Cyclin D1, which promotes cancer cell resistance to ER-targeting drugs. Our previous studies have suggested that the level of reactive oxygen species (ROS) is higher in tumor cells, thus causing oxidation in the nucleotide pool. For hydrolyzing oxidative substrates, NUDT5 activity might be increased.

## Conclusion

Our data indicated that NUDT5 overexpressed in breast tumors. Patients with high NUDT5 expression have bad prognosis. Proliferation, migration, invasion, AKT phosphorylation at Thr308 and Cyclin D1 expression of breast cancer cell lines are enhanced by NUDT5 expression. Thus, NUDT5 may be a novel prognostic factor and potential therapeutic target in breast cancer.

## Supporting information

S1 Fig AKT phosphorylation at Ser473 is inhibited in T47D cell line but not in MCF7 and MDA-MB-231 cell lines. The activation of AKT phosphorylation at Ser473 is inhibited in NUDT5 knock down T47D cell line but not in NUDT5 knock down MDA-MB-231and NUDT5 knock down or re-expressing NUDT5 MCF7 cell lines.
(TIF)

S1 Raw images (PDF)
