# Supplementary figures and images for: The high expression of NUDT5 indicates poor prognosis of breast cancer by modulating AKT / Cyclin D signaling
Source: PLoS One. 2021 Feb 11;16(2):e0245876. doi: 10.1371/journal.pone.0245876 (PMC7877577; doi:10.1371/journal.pone.0245876)

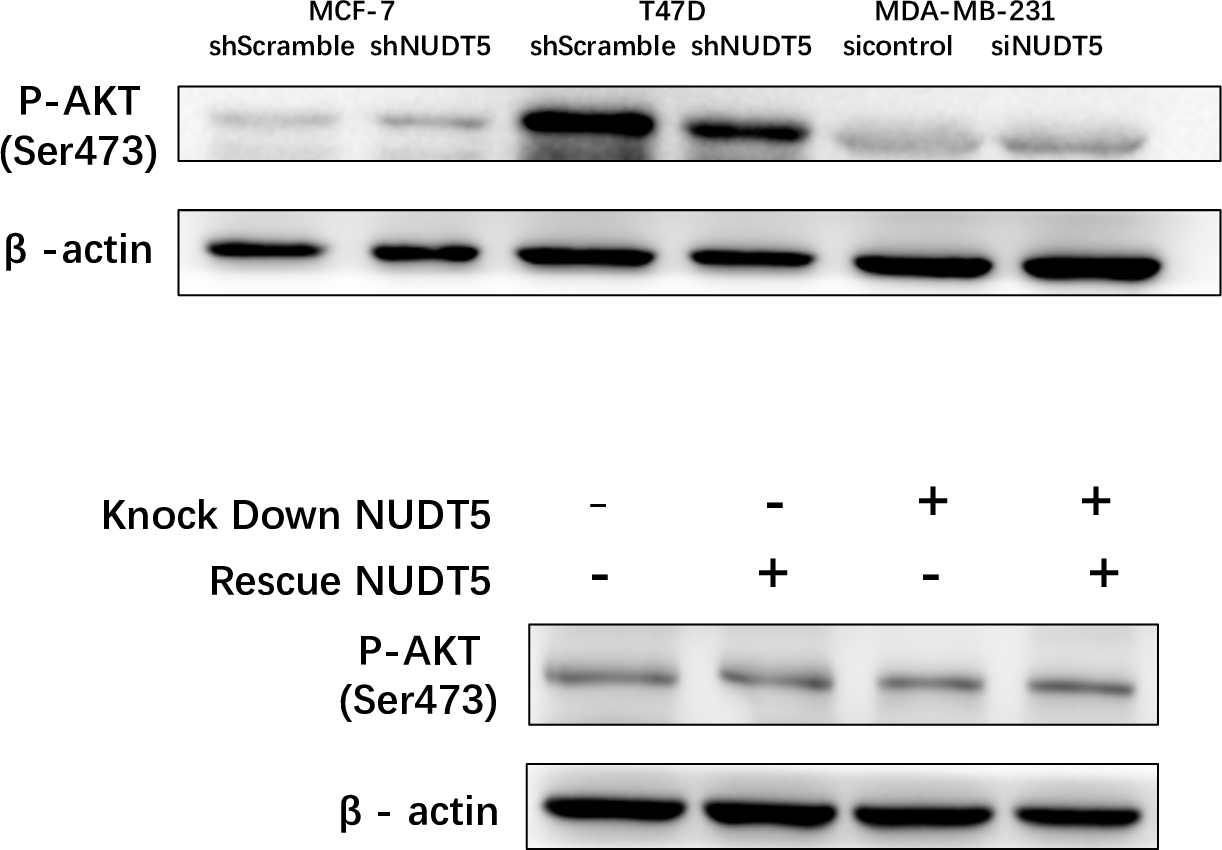

Supplement: S1 Fig — The activation of AKT phosphorylation at Ser473 is inhibited in NUDT5 knock down T47D cell line but not in NUDT5 knock down MDA-MB-231and NUDT5 knock down or re-expressing NUDT5 MCF7 cell lines. (TIF) [file pone.0245876.s001.tif]
