# Supplementary material for: The high expression of NUDT5 indicates poor prognosis of breast cancer by modulating AKT / Cyclin D signaling
Source: PLoS One. 2021 Feb 11;16(2):e0245876. doi: 10.1371/journal.pone.0245876 (PMC7877577; doi:10.1371/journal.pone.0245876)

Figure 3 β -actin

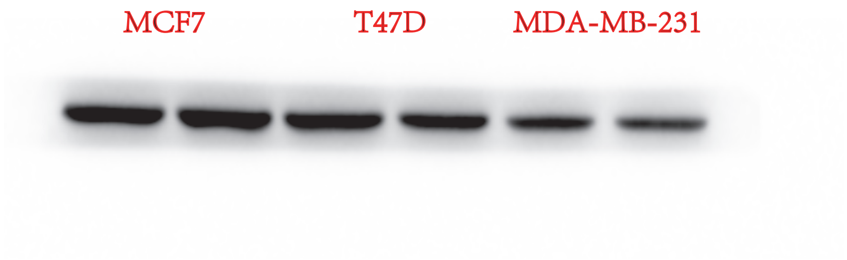

Figure 3 NUDT5

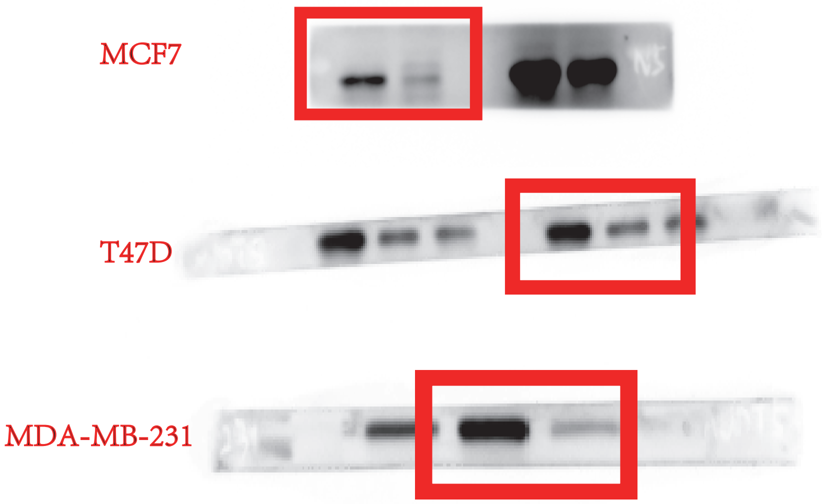

Figure 5 β -actin

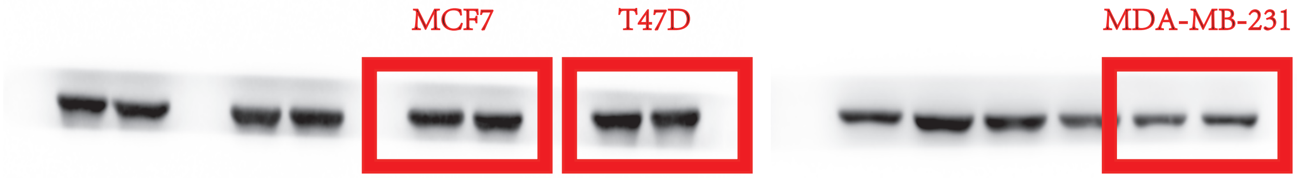

Figure 5 NUDT5

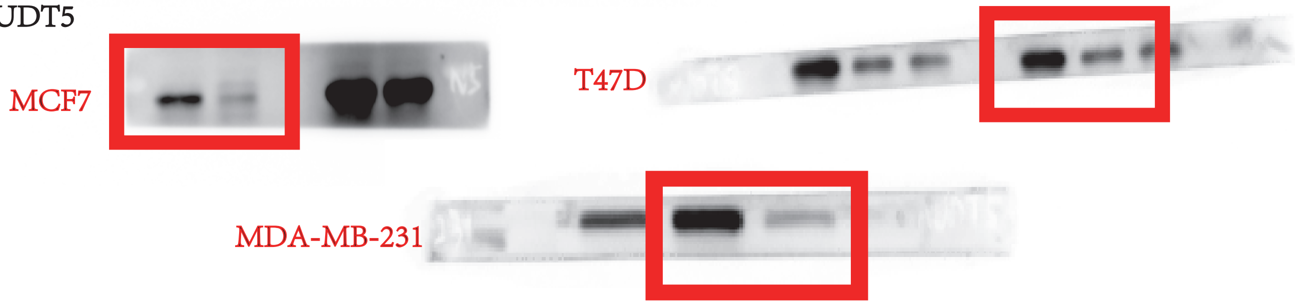

Figure 5 ERK

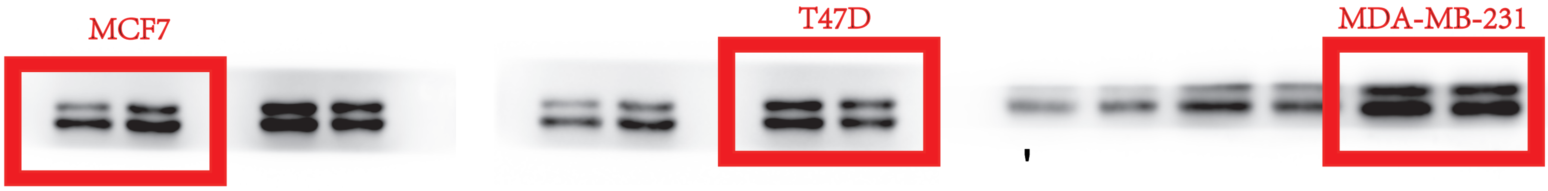

Figure 5 P-ERK

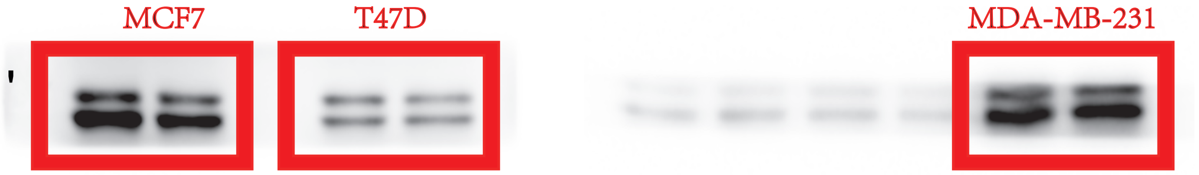

Figure 5 AKT

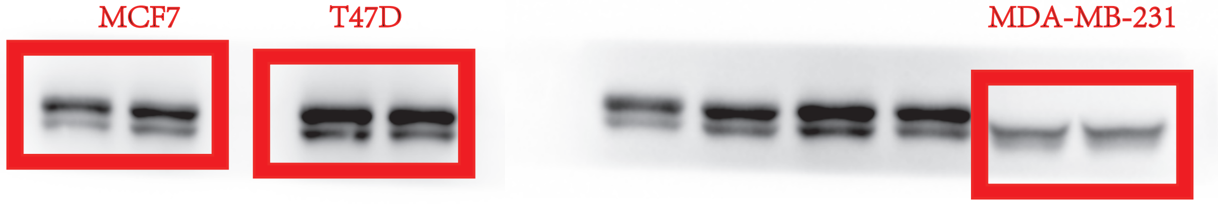

Figure 5 P-AKT

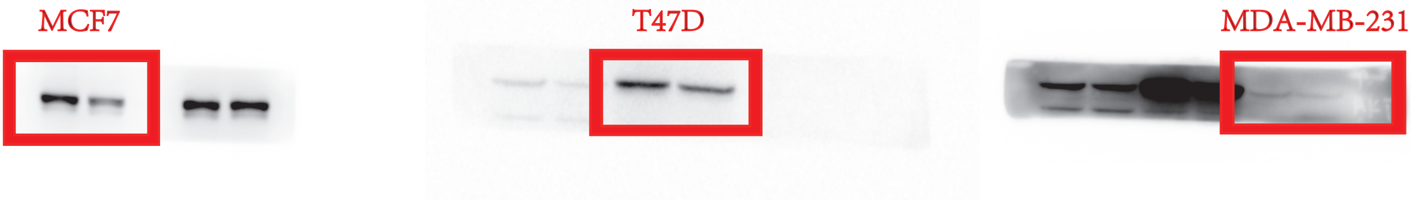

Figure 5 CYCLIND1

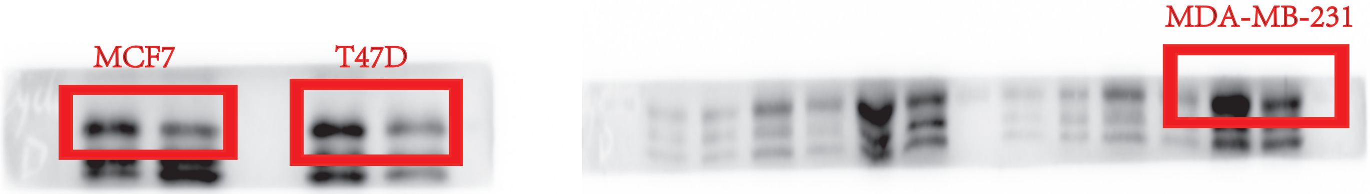

Figure 6 β -actin

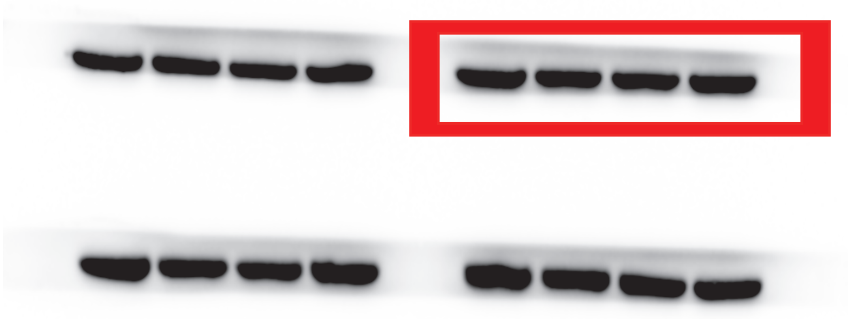

Figure 6 NUDT5

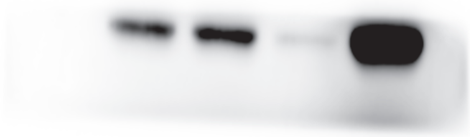

Figure 6 ERK

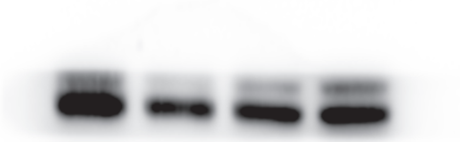

Figure 6 P-ERK

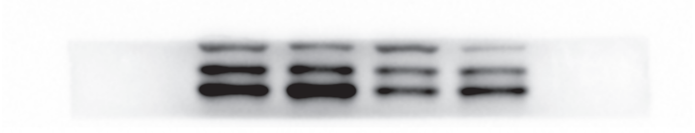

Figure 6 AKT

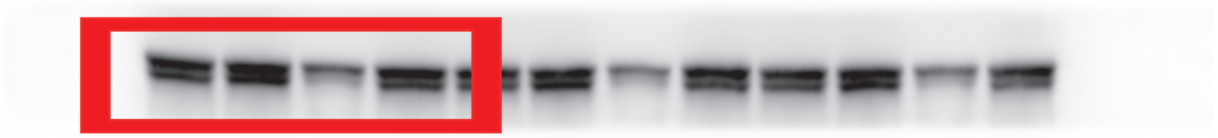

Figure 6 P-AKT

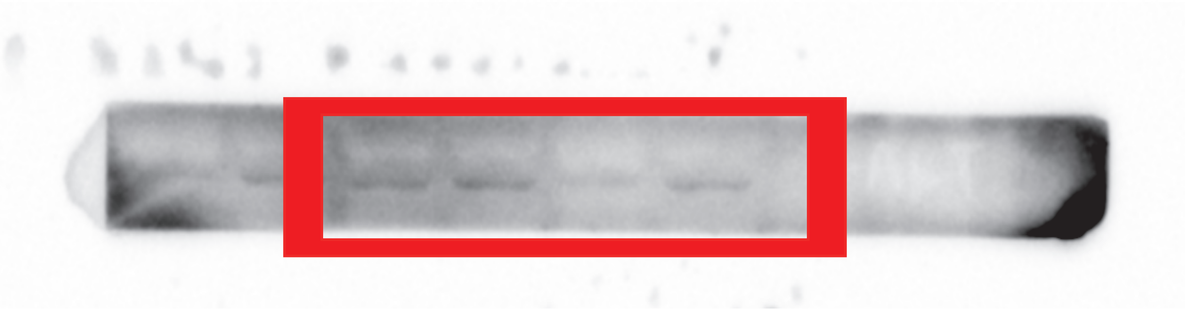

Figure 6 CYCLIND1

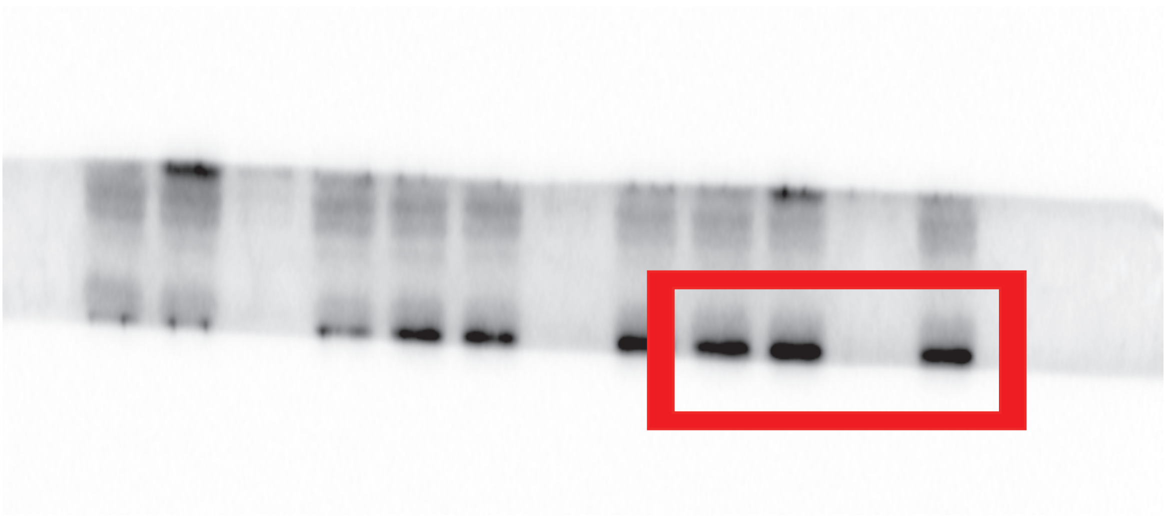

Supplement: S1 Raw images — (PDF) [file pone.0245876.s002.pdf]
